# Physical Improvement and Technical Training

**Published:** 1888-05-26

**Authors:** 


### PHYSICAL IMPROVEMENT AND TECHNICAL TRAINING.

"The first requisite to success in life," says Herbert Spencer, "is to be a good animal." The second, we think, is to be a well-trained animal—an animal whose physical efforts are guided by a well-informed and active mind. The man who possesses these qualities in combination is likely to make his life profitable both to himself and the world. Hitherto we have had—alas! we still have—an enormous number of men who fulfilled only half these requirements—minds with no body to speak of, and bodies with no minds worth counting. We have men who could solve the most difficult problem in Euclid but could not run a hundred yards without being exhausted, and men who could knock you down with the greatest ease, but would be wholly unable to do anything for the broken bones that might result therefrom. These are only half men, incomplete, not the "pure and perfect animal," which by virtue of its perfection is something far more than animal merely. Worse than that, we have had men who in the sense we speak of are hardly men at all—dull minds dwelling in feeble bodies; our large cities breed them in thousands. Our gallant young "barbarians," as Matthew Arnold called them, of the aristocracy, with their muscles developed by rowing, riding, and all manly sports; and the quick-witted, thoughtful scions of the professional classes, with their natural powers increased by a university education—these unite in looking down with a scorn that may be unkind, but is not unnatural, upon the poor "counter-jumper," who can rival neither of them.

Poor counter-jumper! It is true that your highest mental feat may be to calculate the price of three-quarters of a yard of stuff at elevenpence three farthings a-yard, your most violent physical effort—and I confess it is no trivial one—to stand behind a counter looking alert and smiling when your feet are aching with fatigue. It is true that if you are caught in a shower you are apt to develop a pleurisy, and that not infrequently your mental recreation is the reading of such poisonous novels as are apt to lead you only down to sin or suicide; but what chance have you ever had of learning better things?

And the young apprentice, who is set to his life's work while still suffering from the mental dyspepsia that sometimes results from board-school training, his case is a sad one too. He has learnt much, but, like Sir Joseph Porter, has "never thought of thinking for himself at all;" and he sets about his carpentering or engineering with as little interest as he felt n learning the sum of nine times seven or the height of Mount Popocatapetl. It's something he has got to do, must do under certain penalties, but which he does not love, and will shirk and scamp whenever he can. Human machines are made so, but not men. Is it strange that his work should be poor and untrustworthy? His fingers have been put to it but not his soul, and only those tasks are well done to which a man's whole nature, mental, moral, and physical, is conscientiously and lovingly applied.

What is the result of this? To the individuals a dreary, monotonous life, so fatiguing in its dullness that the public-house and the low music-hall offer a welcome excitement—the best they are capable of appreciating. To the nation, the degradation of those in whose hands the balance of electoral power, the destiny of our kingdom, is placed; and—which is a more obvious, and, therefore, to some eyes, a more important consequence—the loss of trade; because foreign workmen, dwellers in countries in which the machinery system is less complete than in England, surpass ours in all but the lowest kinds of labour. Even those who looked upon Ruskin as a weak sentimentalist when he protested that the poor quality and lack of originality in English work was not compensated for by any perfection of finish, begin to think that there is 'something in it" when they accidentally purchase knives that won't cut and boxes with lids that will not hold to the hinge. Whose fault is it? The workman's, for he has no heart for his work, and therefore no conscience in it. The overseer or the employer is supposed to have a monopoly of this commodity; but a collective conscience of that sort is a very inferior thing to the individual possession.

This is but a statement of an old complaint, not worth repeating if there is no remedy. But there is a remedy, and one that may be stated very simply. Give these young men, and now that girls are pressing their claims to share in national labour and national life we may add give these young women, a chance of increasing their culture, and learning more thoroughly the work they have undertaken for their life task. The two things go together, as closely connected as flower and root; the one springs from the other, for it is the application of intelligence to labour that raises it above mere mechanism—gives it that touch of individuality which makes it valuable both intrinsically and in the open market; for purchasers are as ready to give a high price for work that bears evidence of thought and taste as to demand that machine work—the things that can be turned out by the score—shall be supplied to them as cheaply as possible.

In other words, establish technical schools, and in connexion with these provide classes where the pupils may gain a real knowledge of some of those arts which "engentle humanity," and those exercises which strengthen the intellect.

But we have said that this mental training is incomplete without physical development; indeed, it cannot proceed far without it. When the body is weak it cannot, as a rule, stand the strain of mental growth; therefore the ideal technical school must have connected with it a gymnasium and other means of physical improvement and recreation. Lads who have been cooped up in shops and warehouses all day want this as much as opportunities for further study; indeed, till the congested blood has got into the habit of flowing more swiftly through their bodies, till their brains are clear and their eyes bright, they will not profit much by all the studies you could bring before them.

This is no small thing to establish in a city like London, where everything, the needs of its population among the rest, is on a gigantic scale. But it has been done; the People's Palace is an active proof of it; but the People's Palace supplies the wants of only one quarter of London, and the other districts plead their like necessities. The East End, as a newly-discovered country, has attracted a perhaps disproportionate share of public interest and alms. A lady once aid to me that while she could get a score of friends to help her in any scheme for the benefit of our London Orientals, it was difficult to rouse a spark of interest in a West End charity. The Mrs. Jellyby of these days finds her Borioboola Gha in the wilds of Stepney and Whitechapel, and forgets the wants of the attentive "young persons" in her favourite draper's, and of the dwellers in those shabby little streets, with a flavour of mews about them, that lurk behind the big houses and shops of the West.

It certainly has been left for the munificence of one man, Mr. Quintin Hogg, aided by his energetic secretary, Mr. R. Mitchell, to provide a club, gymnasium, and school for the working men and women of the West End. For twenty-four years, ever since he left Eton a youth himself, he has given all his spare time to helping youths less fortunately placed than he was. He began with a ragged schoo in the neighbourhood of Drury Lane, and this was soon developed into a home for working boys. In 1873 there was added to this an institute in Endell Street, midway between Drury Lane and Seven Dials, which was established "for the purpose of endeavouring to withdraw elder boys from evil surroundings." This prospered and grew, and soon was transferred to larger premises in Long Acre. Even these were found too small for all the boys needed and all Mr. Hogg desired to do, and when six years ago the Polytechnic Institution was offered for sale, he secured it for a further development of his Institute. The Polytechnic's day had gone by. Its popular science was no longer popular, only thin; the school children of our generation would not wonder at its marvels, they knew how they were produced, and gazed with indifference, not awe. The Polytechnic was going a-begging when Mr. Hogg bought it, and put it to a wider use than it had ever dreamed of. The diving-bell collapsed in a new swimming bath, which a writer in the *Daily News* declared to be the handsomest in London. This bath exists, however, in summer only; at other seasons it is drained, carpeted, and comfortably furnished, and becomes the chief reading room of the place.

Besides this there are other, permanent, reading-rooms, rooms for chess and draughts, a hall for the rhetoric of the Debating Society, and a large gymnasium where the athletic members of the Institute disport themselves with grace and vigour in feats that make the onlooker tremble.

As for the societies devoted to healthy amusement in connexion with the institute, they embrace all that the mind of young and vigorous manhood can desire. There are athletic, cricket, football, and lawn tennis clubs, that play on the ground at Merton Hall, Wimbledon, the largest cricket ground in England, extending to 27 acres. There is a rowing club, a rambling club, a harriers' club, and a gymnastic club. The Polytechnic supplies the 4th Middlesex Volunteer Company, and a Medical Staff Corps attached to it; a Polytechnic battery to the 1st City of London Artillery, and a Polytechnic Company to the 1st Middlesex Engineers. The musical members may belong to the Military, the Concertina, or the Fife and Drum Band; the Orchestral Society, or the Choral Society. Those whose tastes lead them in other directions may study elocution, join the Debating Society, or spend their evenings in acquiring French, German, geometry, or shorthand.

All this is apart from the technical training, which is very thorough, and is limited by only one condition beyond the payment of a small fee—namely, that the student who wishes to profit by them must be already engaged on the trade he means to study. The Polytechnic does not wish to turn out amateur carpenters, photographers, wood-carvers, and so forth; there are enough and to spare of these already. The managers wish to help industrious workmen to perfect themselves in their trades, to fit them, while they are still earning their living by the elementary branches of their business for doing good work in it when the opportunity comes. It is impossible to give a complete list of all these classes; they embrace almost every industry of civilised life; and how thoroughly they are appreciated is shown by the numbers of young men who gather in the workshops in the evenings between seven and ten o'clock, and the earnest look on their faces as they listen to their teachers or make experimental attempts in the higher branches of their vocations—their carriage-building, or tool-making, tailor's cutting, watchmaking, or upholstery.

For the young women there are special classes in cookery, dressmaking, and millinery, and they may share in the instruction in the general subjects, those not confined to special trades, and thus gain a knowledge of the piano, violin, or harmonium, French, book-keeping, and shorthand.

How much these opportunities are appreciated is shown by the fact that last session the students numbered 6,500, and contributed £9,000 to the support of the institution. The cost of maintenance, however, amounts to about £15,000, and hitherto the deficit has been entirely paid by Mr. Quintin Hogg, who, since he began his work for the lads of London, has spent about £100,000 on it. He is anxious now to see the institute so provided for that it can go on in the event of his death without being cramped for lack of means. For this purpose he has applied to the Charity Commissioners for an endowment for the Polytechnic. These have promised to give £2,500 per annum on condition that a sum of £35,000 be raised as a supplementary fund. Mr. Hogg's personal friends have already promised £18,000 of this; he now asks the public for the remaining £17,000.

Surely he will not ask in vain. It is everybody's interest to help our young men and women to acquire greater physical strength, intellectual culture, and technical training; it is they who, according as they work ill or well, according as they have thoughtful, balanced minds or the reverse, will make or mar the destinies of our nation. Surely it is a duty to give them the opportunity for becoming better, wiser, braver, stronger, and not the opposite of all these things; surely it is well that their natural, healthy desire for amusement should be gratified in such a way that when they utter the prayer, "Lead us not into temptation," it shall not be as a vain cry of despair.

Some people say such an institution should be self-supporting. How many of our good educational institutions are self-supporting? Not our great schools, not our universities. These all owe their prosperity and their possibilities of usefulness to the endowments derived from long-dead benefactors; it is not likely that this university for workmen, which requires not only skilled teachers, but elaborate and often expensive machinery, should be able all at once to pay its expenses. The students do well when they give, out of incomes that are very limited indeed, nearly two-thirds of the necessary revenue; surely generous London, that profits now and will in the future profit more and more by the thorough training in their work these apprentices and others are trying to obtain, will not refuse to give the rest.

